# Isolated nasal septal involvement in granulomatosis with polyangiitis: Radiologic features and literature review

**DOI:** 10.1016/j.radcr.2026.04.071

**Published:** 2026-05-25

**Authors:** Mohamed Bouallou, Achraf Amine Sbai, Issam Berrajaa, Drissia Benfadil, Azeddine Lachkar

**Affiliations:** a Department of Otorhinolaryngology, Mohammed VI University Hospital, Oujda, 60000, Morocco; b Faculty of Medicine and Pharmacy, Mohammed First University, Oujda, 60000, Morocco

**Keywords:** Granulomatosis with polyangiitis, Nasal septum, Septal perforation, Computed tomography, ANCA

## Abstract

Granulomatosis with polyangiitis is a necrotizing granulomatous vasculitis that predominantly involves the upper and lower respiratory tracts and the kidneys. Although sinonasal manifestations are common, they are often nonspecific and can mimic benign inflammatory conditions, leading to delayed diagnosis. In contrast, strictly localized disease limited to the nasal septum without systemic involvement is exceptionally rare. We report the case of a 27-year-old woman presenting with persistent nasal obstruction and rhinorrhea. Nasal endoscopy revealed a septal perforation. Computed tomography demonstrated focal nasal septal lysis without paranasal sinus involvement. Laboratory investigations showed markedly elevated c-ANCA levels, and histopathological examination of a septal biopsy confirmed granulomatosis with polyangiitis. Systemic imaging revealed no pulmonary or renal involvement, supporting a localized form of the disease. The patient was treated with systemic corticosteroids and methotrexate, resulting in clinical remission at 5 months. This case highlights the crucial role of imaging in detecting early structural abnormalities and guiding biopsy in atypical presentations of GPA. Early recognition of localized disease is essential to initiate appropriate immunosuppressive therapy and prevent progression to systemic involvement.

## Introduction

Granulomatosis with polyangiitis is a rare, potentially life-threatening necrotizing granulomatous vasculitis affecting small- to medium-sized vessels. It classically involves the upper and lower respiratory tracts as well as the kidneys, but its clinical spectrum is highly heterogeneous, ranging from localized disease confined to the ENT region to rapidly progressive systemic involvement [1].

Sinonasal manifestations are common and often represent the earliest clinical presentation. However, they are frequently nonspecific and may mimic benign inflammatory or infectious conditions, leading to diagnostic delay [2]. From a radiological perspective, CT plays a pivotal role in detecting early structural abnormalities, assessing osseocartilaginous destruction, and guiding targeted biopsy.

Strictly localized disease limited to the nasal septum, without sinonasal extension or systemic involvement, remains exceptionally rare and poorly described in the literature [3]. Such presentations pose significant diagnostic challenges, particularly in young patients and in the absence of classical systemic features.

We report an unusual case of GPA revealed by isolated nasal septal perforation in a young woman without pulmonary or renal involvement. This report underscores the crucial contribution of imaging in raising diagnostic suspicion, discusses relevant differential diagnoses of septal perforation, and provides a focused review of previously published cases to better delineate this uncommon presentation.

## Case presentation

We report the case of a 27-year-old female patient with no significant past medical history, who was referred to our Department of Otolaryngology for evaluation of persistent unilateral nasal obstruction associated with rhinorrhea. There was no history of diabetes mellitus, craniofacial trauma, prior endonasal surgery, or illicit drug use.

The symptoms had an insidious onset approximately 3 months prior to presentation, characterized by progressive nasal obstruction and anterior rhinorrhea. The patient was initially managed in primary care with topical and systemic corticosteroids combined with isotonic saline nasal irrigation, without clinical improvement, prompting referral to our tertiary care center.

**Fig. 1:**
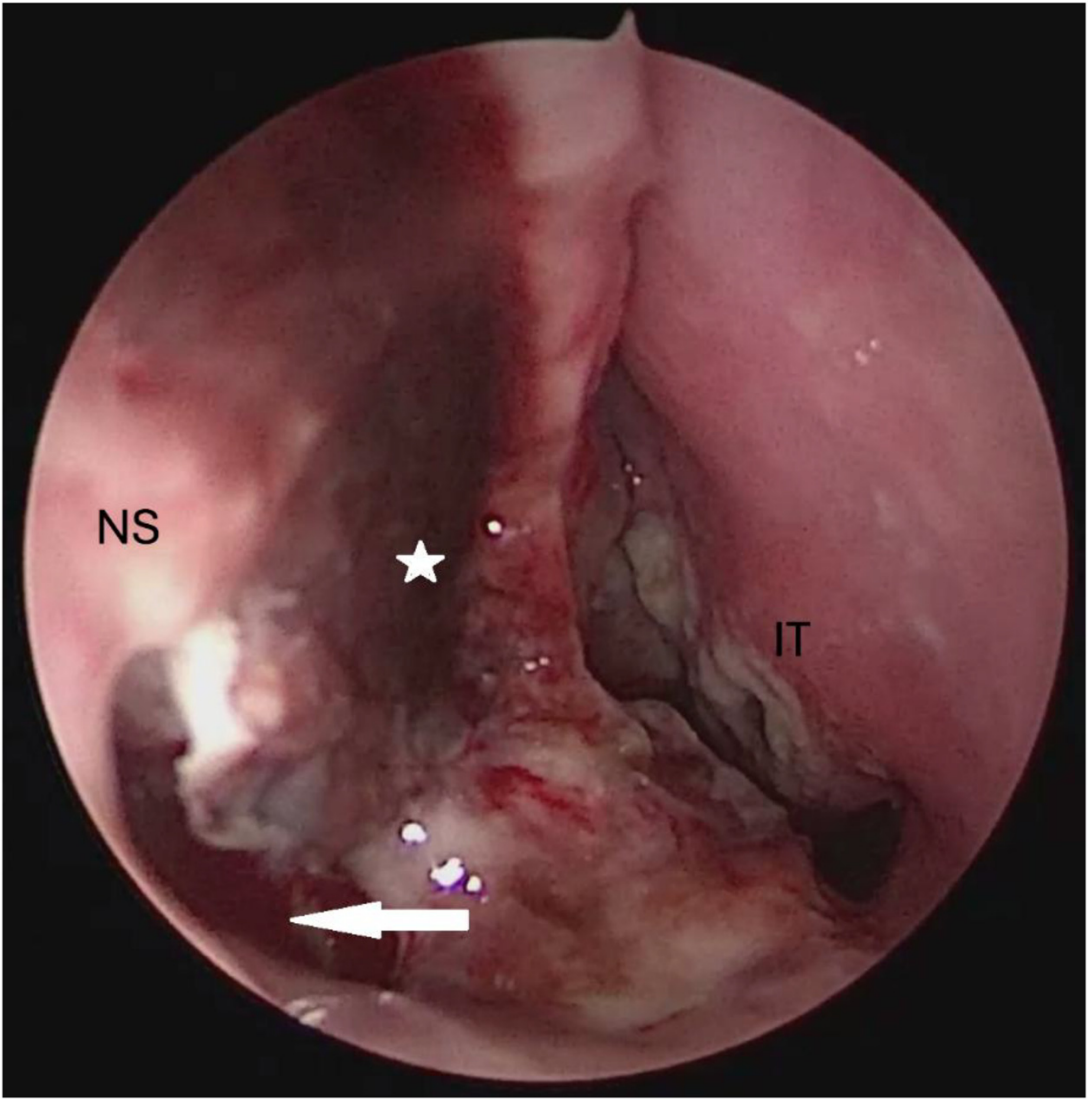
Left anterior rhinoscopy demonstrating a septal perforation (white arrow) associated with septal deviation, necrotic septal mucosa (white star), and atrophy of the inferior turbinate. **IT,** inferior turbinate; **NS,** nasal septum.

On general examination, the patient was alert, afebrile, and hemodynamically stable, with no respiratory distress. Nasal endoscopy revealed inflamed nasal mucosa, bilateral inferior turbinate atrophy, right-sided septal deviation, and a septal perforation (Fig. 1). Cervical examination revealed no palpable lymphadenopathy, and the remainder of the head and neck evaluation was unremarkable. Pulmonary examination was also normal.

**Fig. 2:**
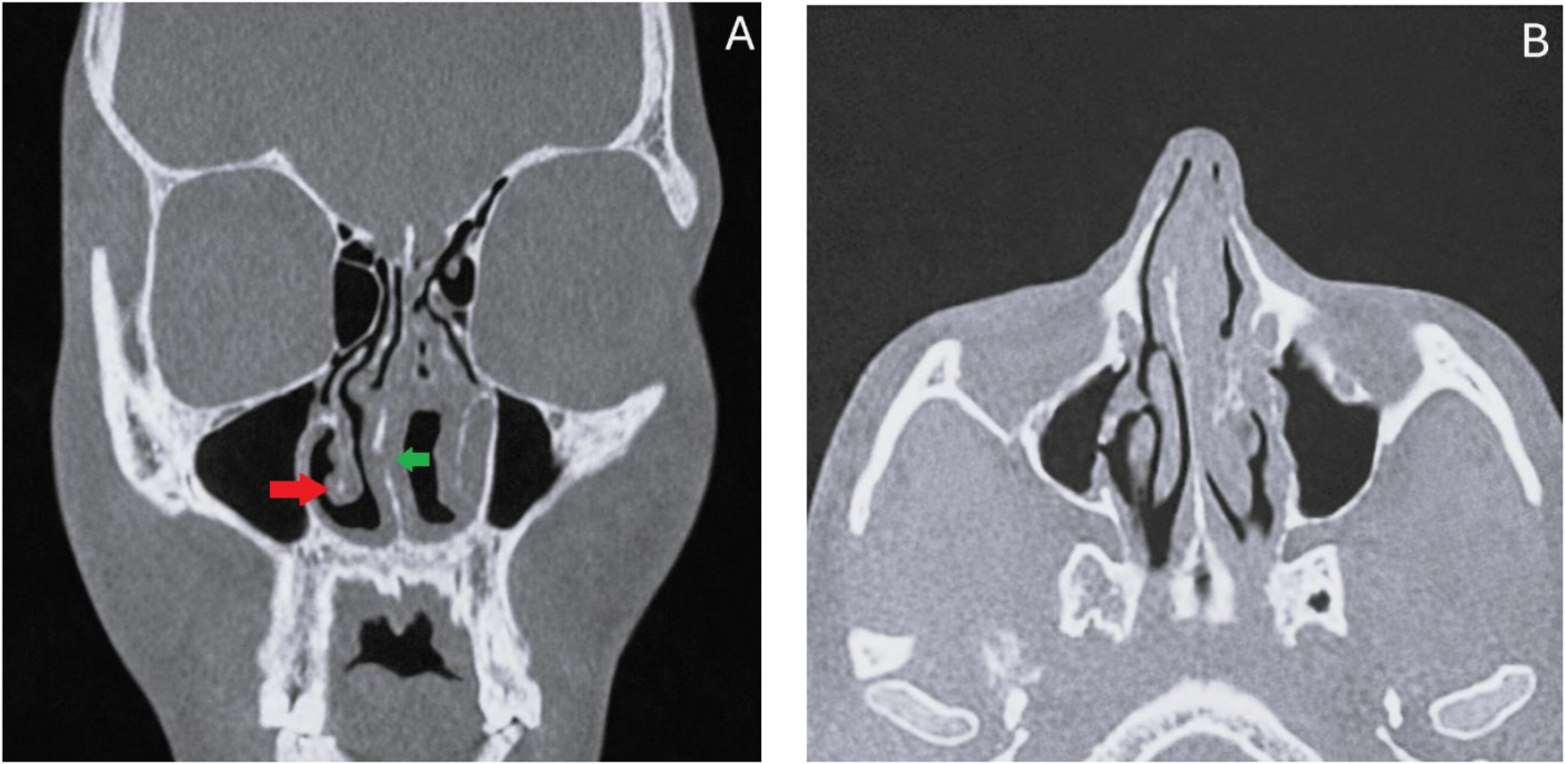
**(A)** Coronal head and neck CT scan (bone window) demonstrating a well-defined lytic defect involving the cartilaginous portion of the nasal septum (green arrow), consistent with focal septal lysis, associated with bilateral inferior turbinate atrophy (red arrow). The margins of the septal defect appear irregular but clearly delineated, without extension to the bony septum. **(B)** Axial contrast-enhanced CT scan showing leftward septal deviation with focal thickening of the septal mucosa. No abnormal soft tissue mass or contrast enhancement is identified.

A contrast-enhanced head and neck computed tomography scan demonstrated right-sided septal deviation associated with focal septal lysis. The paranasal sinuses were clear, with associated inferior turbinate atrophy (Fig. 2).

**Fig. 3:**
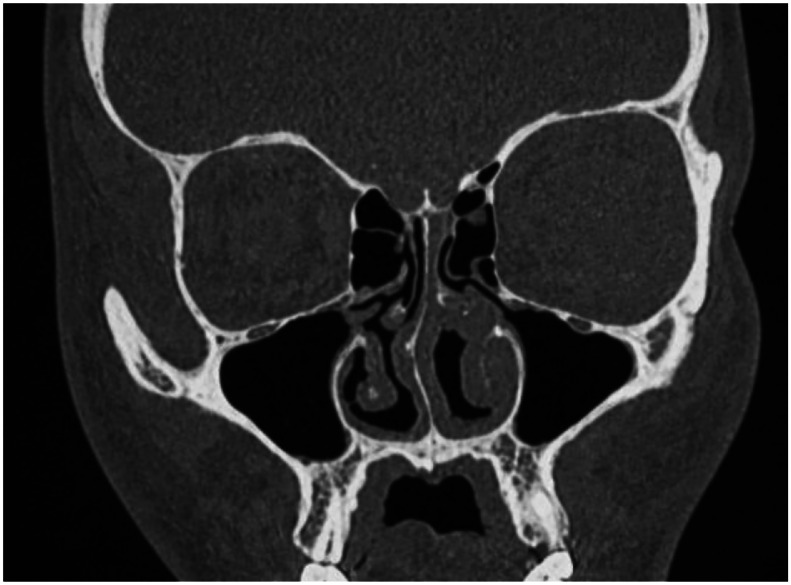
Non-contrast coronal CT scan (bone window) demonstrating a slight reduction in the size of the nasal septal perforation compared with the initial examination. No progression of osseous destruction is observed.

A follow-up contrast-enhanced CT scan was performed 4 months after treatment initiation. It demonstrated a slight reduction in the size of the nasal septal perforation, without progression of osseous destruction or development of new sinonasal abnormalities, consistent with radiological stability under treatment (Fig. 3).

Laboratory investigations revealed marked leukocytosis (WBC: 18.9 × 10⁹/L [normal: 4-10 × 10⁹/L]) with neutrophilia (85.7% [normal: 40%-75%]), and an elevated erythrocyte sedimentation rate (39 mm/h [normal: <20 mm/h]). Serological testing for HIV, hepatitis B, and hepatitis C viruses was negative. Notably, cytoplasmic anti-neutrophil cytoplasmic antibodies (c-ANCA) were markedly elevated (>355 U/mL [normal: <10 U/mL]). Urinalysis was normal, with no proteinuria or hematuria. Renal and hepatic function tests were within normal limits.

**Fig. 4:**
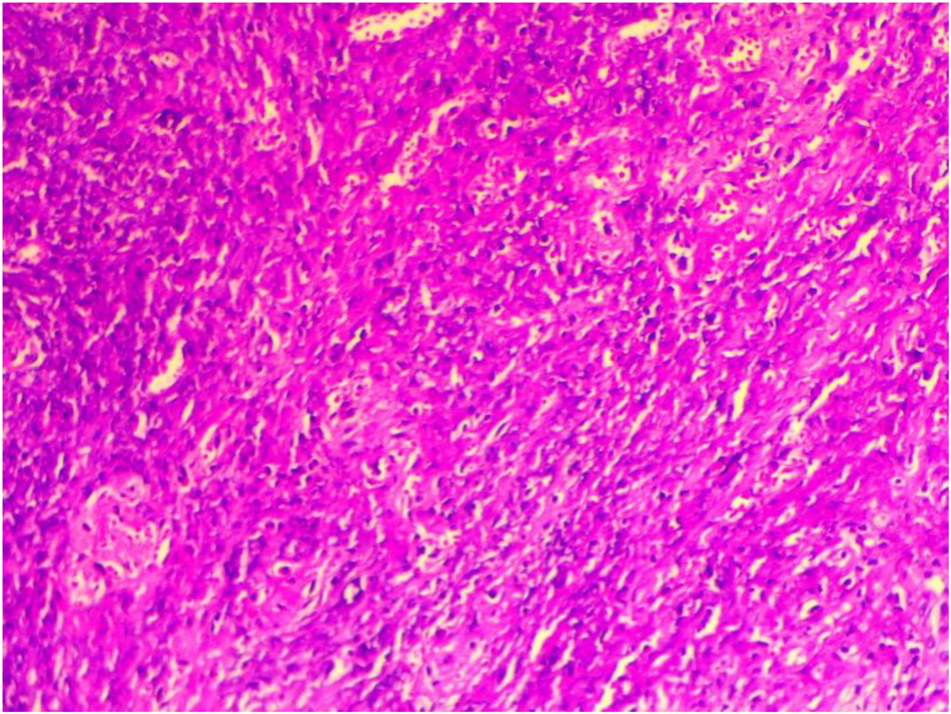
Histopathological image showing ulcerated nasal mucosa with a diffuse chronic inflammatory infiltrate associated with areas of necrosis and multinucleated giant cells forming granulomas surrounded by plasma cells and lymphocytes.

A biopsy of the nasal septum was performed using a 30° rigid endoscope. Histopathological examination revealed granulomatous inflammation with focal necrosis and vasculitis, thereby confirming the diagnosis of granulomatosis with polyangiitis (Fig. 4).

A comprehensive cervico-thoraco-abdomino-pelvic CT scan, including bone and parenchymal windows as well as lung window analysis, showed no evidence of systemic involvement.

The patient was managed in collaboration with the internal medicine department. Treatment consisted of systemic corticosteroids (prednisone 1 mg/kg/day for 3 months), methotrexate (15 mg/week for 6 months), antibiotic prophylaxis with co-trimoxazole (trimethoprim–sulfamethoxazole), and topical nasal corticosteroids. Clinical follow-up was conducted every 3 weeks with nasal endoscopy. Disease remission was achieved at approximately the fifth month.

Surgical repair of the septal perforation was deferred until sustained disease remission is achieved.

## Discussion

Granulomatosis with polyangiitis is a rare necrotizing granulomatous vasculitis involving small- to medium-sized vessels [4]. It classically affects the respiratory tract and kidneys. However, its clinical presentation is highly heterogeneous, ranging from localized ENT-limited disease to fulminant systemic involvement. Otorhinolaryngological manifestations represent the initial presentation in more than 70% of patients, and they are frequently misdiagnosed as infectious or allergic conditions [2]. This case illustrates a misleading presentation initially interpreted as simple rhinitis, contributing to diagnostic delay.

GPA typically affects individuals between the fourth and sixth decades of life, without a clear sex predilection [5]. Its pathogenesis is thought to result from the interplay between genetic susceptibility and environmental triggers, including chronic nasal colonization by *Staphylococcus aureus*, exposure to silica, and certain pharmacological agents [6]. The localized form, typically confined to the upper and/or lower respiratory tract without renal involvement, accounts for approximately 10% of cases at diagnosis [7]. Strictly isolated nasal septal involvement, as observed in our patient, remains exceptionally rare, with only sporadic cases reported in the literature and no precise estimate of its prevalence.

To better contextualize our findings, previously reported case reports describing isolated nasal septal involvement in GPA are summarized in Table 1.

**Table 1:** Reported cases of granulomatosis with polyangiitis presenting with nasal septal involvement.

Study	Age/Sex	Clinical presentation	Type of septal involvement	Treatment	Key features
Sumaily et al. [8]	32 M	Destructive nasal mass	Septal destruction	Cyclophosphamide + corticosteroids	Mimicked neoplastic lesion
Ralli et al. [9]	50 F	Nasal mass with perforation	Septal perforation	Cyclophosphamide + corticosteroids	Tumor-like presentation
Sasawaki et al. [10]	63 M	Septal abscess	Multifocal septal abscess	Corticosteroids + immunosuppressants	Rare abscess-like presentation
Maqbool et al. [11]	45 F	Nasal obstruction, crusting	Septal perforation	Cyclophosphamide + corticosteroids	Initially misdiagnosed
Enabi et al. [3]	68 F	Localized nasal bridge involvement	Localized septal/bridge disease	Corticosteroids + methotrexate	Limited ENT form
Chabane Sari Mehtari et al. [12]	38 F	Complete septal destruction	Extensive septal perforation	Cyclophosphamide + corticosteroids	Advanced ENT disease
Our case (2026)	27 F	Isolated septal perforation with crusting and obstruction	Localized septal perforation	Corticosteroids + methotrexate + cotrimoxazole prophylaxis	Early-stage localized GPA, remission in 5 months

Compared with previously published case reports of GPA involving the nasal septum, our case shares key diagnostic features, notably c-ANCA positivity and septal destruction. However, it is distinguished by its strictly localized presentation without pulmonary or renal involvement and by the relatively young age of the patient. Most reported cases involve older individuals presenting at a more advanced stage, frequently with established saddle-nose deformity.

In contrast, our patient was diagnosed at an earlier stage of disease and achieved complete remission under a less intensive therapeutic approach combining systemic corticosteroids and methotrexate. These findings underscore the critical importance of early recognition, particularly in younger patients, to prevent progression toward irreversible structural damage and to allow effective disease control with tailored, less toxic treatment strategies. This observation further suggests that early-stage, localized GPA, particularly in younger patients, may follow a more favorable clinical course when promptly recognized and appropriately treated.

Clinically, sinonasal GPA frequently mimics chronic rhinosinusitis. Patients typically present with persistent nasal obstruction, mucopurulent discharge, crusting, and recurrent epistaxis that are often resistant to standard medical therapy [13]. Ongoing necrotizing inflammation may progressively destroy the nasal septum, leading to septal perforation and, in advanced stages, saddle-nose deformity secondary to cartilaginous collapse. In our patient, the clinical presentation was nonspecific, consisting of rapidly progressive nasal obstruction and rhinorrhea over a 3-month period, with extensive crusting that initially masked the septal perforation.

Nasal septal perforation in GPA results from the combined effects of necrotizing small-vessel vasculitis and granulomatous inflammation involving the vascular supply of the septal cartilage. ANCAs, particularly anti–proteinase 3 (PR3), induce neutrophil activation and subsequent endothelial injury through the release of reactive oxygen species, proteolytic enzymes, and proinflammatory mediators, leading to ischemic damage of the perichondrium, which is critical for cartilage nutrition [4]. Concomitantly, an imbalance between matrix metalloproteinases and their tissue inhibitors promotes extracellular matrix degradation, thereby weakening septal structural integrity and culminating in cartilage necrosis and perforation, as observed in our patient.

In the present case, the disease was strictly confined to the nasal septum, with no evidence of pulmonary, renal, or systemic involvement on comprehensive imaging and laboratory assessment. The clinical presentation was nonspecific, characterized by rapidly progressive nasal obstruction and rhinorrhea over a 3-month period, with the presence of crusting masking the septal perforation, which significantly contributed to diagnostic uncertainty.

From a biological perspective, serological testing for ANCA is essential for establishing the diagnosis. Cytoplasmic ANCA (c-ANCA) demonstrates a sensitivity of approximately 90%-95% in active generalized forms of GPA, but this sensitivity decreases to around 60% in early-stage or localized disease [14]. Positive ANCA results should be further characterized by anti-proteinase 3 (PR3) and anti-myeloperoxidase (MPO) antibodies, which provide higher diagnostic specificity. In our case, the patient exhibited markedly elevated c-ANCA levels with high titers of anti-PR3 antibodies, findings that strongly supported the diagnosis despite the localized presentation.

Histopathological examination remains the diagnostic gold standard for GPA, classically revealing necrotizing vasculitis, granulomatous inflammation, and multinucleated giant cells [15]. However, the sensitivity and specificity of biopsy vary according to the anatomical site and disease activity, with sinonasal specimens showing a higher rate of false-negative results compared with other involved organs [14]. To minimize the risk of diagnostic error in our patient, multiple targeted biopsies were obtained from the nasal septum and the inferior turbinate mucosa to increase diagnostic yield, allowing definitive histopathological confirmation.

Imaging plays a fundamental role in the evaluation of sinonasal involvement in GPA. CT is particularly useful for assessing mucosal disease, identifying structural damage, and evaluating the integrity of the nasal septum and surrounding osseocartilaginous structures.

A systematic review by D’Anza et al. [16] demonstrated that mucosal thickening represents the most frequently reported finding on computed tomography in sinonasal GPA, whereas septal erosion was observed far less commonly. A recent retrospective cohort study by Tateyama et al. [17] including 17 patients with granulomatosis with polyangiitis, demonstrated that mucosal thickening and bony hypertrophy were the most prevalent radiological findings, observed in 94.1% and 70.6% of cases, respectively, whereas bone destruction was identified in only 23.5% of patients.

The absence of paranasal sinus involvement in our patient further highlights the atypical and strictly localized nature of the disease. Such an imaging pattern remains uncommon and underscores the necessity of correlating radiological findings with clinical presentation and serological markers to achieve diagnostic accuracy. In this context, computed tomography not only enables precise characterization of structural alterations but also facilitates targeted biopsy by identifying the most representative sites of disease involvement. Moreover, thoracic imaging plays a complementary role in the evaluation of systemic disease, particularly in detecting pulmonary manifestations such as nodules, cavitations, or ground-glass opacities suggestive of diffuse alveolar hemorrhage [18].

Although magnetic resonance imaging was not required to evaluate systemic extension in our case, MRI may provide complementary information in selected patients, particularly when soft tissue infiltration, orbital extension, skull base involvement, or intracranial complications are suspected [16]. In addition, MRI offers superior soft-tissue contrast resolution compared with computed tomography, allowing a more accurate characterization of inflammatory versus granulomatous tissue, as well as earlier detection of subtle locoregional extension, including perineural spread, orbital infiltration, and intracranial involvement, which may remain occult on CT imaging.

The differential diagnosis of isolated nasal septal perforation is broad and requires structured approach. Infectious causes must be considered first, particularly tuberculosis and syphilis, especially in endemic settings such as Morocco. Non-infectious inflammatory conditions, notably sarcoidosis, may also present with granulomatous involvement of the nasal mucosa and closely mimic vasculitic disorders. Neoplastic etiologies, including extranodal NK/T-cell lymphoma and sinonasal carcinomas, should be carefully excluded given their potential for rapidly progressive and locally destructive behavior. In addition, non-inflammatory causes such as iatrogenic injury, prior trauma, and cocaine-induced midline destructive lesions must be considered, as they can lead to septal perforation through ischemic and necrotizing mechanisms.

From a radiological standpoint, computed tomography plays a key role in narrowing this differential diagnosis. CT imaging allows precise assessment of the pattern of septal destruction, the presence or absence of an associated soft-tissue mass, and the degree of surrounding sinonasal involvement. Aggressive neoplastic processes are typically associated with expansile soft-tissue masses, bone remodeling, or invasion of adjacent structures, whereas infectious diseases may demonstrate mucosal thickening, sinus opacification, or collections suggestive of abscess formation.

In the present case, the relatively rapid progression of symptoms over a 3-month period, along with septal lysis on imaging, initially raised concern for an underlying neoplastic process. However, CT imaging demonstrated a well-defined septal defect without an associated soft-tissue mass, bone remodeling, or aggressive invasive features. The absence of paranasal sinus opacification or fluid collections further argued against an infectious etiology. These radiological characteristics therefore favored an inflammatory or vasculitic process rather than a neoplastic or infectious cause.

The presence of a strongly positive c-ANCA, in conjunction with histopathological findings of necrotizing granulomatous inflammation and vasculitis, ultimately supported the diagnosis of a localized form of GPA.

Therapeutic management of GPA is conventionally divided into an induction phase followed by a maintenance phase. The induction phase aims to achieve rapid disease remission through systemic glucocorticoid therapy, typically administered over 3 to 6 months, in order to control active inflammation and prevent organ damage [12].

Once remission is obtained, maintenance therapy is initiated to reduce the risk of relapse, most commonly using immunosuppressive agents such as methotrexate or azathioprine for a prolonged duration, often exceeding 18 months.

Plasma exchange may be considered in severe systemic forms, particularly in cases complicated by diffuse alveolar hemorrhage, rapidly progressive renal impairment, or overlap syndromes involving anti–glomerular basement membrane antibodies, as observed in Goodpasture syndrome [14].

In localized or non–organ-threatening forms, as observed in our patient, methotrexate combined with glucocorticoids is widely recommended and has demonstrated favorable remission rates exceeding 80%-90% [19,20]. In contrast, more severe or systemic forms typically require more aggressive induction regimens, including cyclophosphamide or rituximab. Both therapies have demonstrated comparable efficacy in achieving remission at 6 months, with remission rates of approximately 64% for rituximab and 53% for cyclophosphamide, as reported in the RAVE trial [21].

In our case, treatment was tailored to the localized nasal septal involvement of GPA. Therapy with corticosteroids and methotrexate achieved remission by the fifth month, with clear endoscopic improvement.

Surgical management does not influence the underlying disease activity in GPA. However, it may play a valuable role in addressing the functional and aesthetic sequelae resulting from tissue destruction. Reconstructive procedures should be carefully timed and are preferably undertaken during phases of sustained remission [13]. Indeed, surgical intervention during active or relapsing disease is strongly discouraged, as it may exacerbate local inflammation, impair wound healing, and ultimately lead to more pronounced structural damage and unfavorable outcomes. In our patient, rhinoseptoplasty was therefore postponed until stable remission was achieved.

These findings suggest that early-stage, localized granulomatosis with polyangiitis, particularly in younger patients, may have a more favorable outcome when promptly diagnosed and treated. The combined use of imaging, serological markers, and histopathology is essential for early detection. Recognizing such atypical presentations allows timely initiation of appropriate immunosuppressive therapy, thereby reducing the risk of irreversible damage and progression to systemic disease.

Nevertheless, the relatively short duration of follow-up in the present case, limited to 5 months, represents a potential limitation. Although clinical remission was achieved during this period, longer-term monitoring remains essential to evaluate the durability of disease control and to detect possible relapse or delayed systemic manifestations, which are well recognized in granulomatosis with polyangiitis. Future studies with extended follow-up may therefore provide a more comprehensive understanding of the long-term evolution of strictly localized nasal forms of the disease.

## Conclusion

Isolated nasal septal involvement in granulomatosis with polyangiitis is an exceptionally rare presentation that may mimic more common inflammatory conditions. Computed tomography plays a key role in detecting early structural abnormalities, characterizing septal destruction, and guiding targeted biopsy. Early recognition of this localized form allows timely initiation of immunosuppressive therapy, potentially preventing irreversible tissue damage and systemic progression.

## Patient consent

The patient gave their informed consent to the publication of this case report.

## References

[1] Patil V., Auradkar A., Muniraju M., Faseeh K.M. (2026). Otolaryngologic manifestations in granulomatosis with Polyangitis: a systematic review of clinical presentations and renal correlation. Eur Arch Otorhinolaryngol.

[2] Carnevale C., Arancibia-Tagle D., Sarría-Echegaray P., Til-Pérez G., Tomás-Barberán M. (2019). Head and neck manifestations of granulomatosis with polyangiitis: a retrospective analysis of 19 patients and review of the literature. Int Arch Otorhinolaryngol.

[3] Enabi J., Sharif W., Mannem M., Rodriguez Vazquez J.L., Mukkera S. (2024). Granulomatosis with polyangiitis (GPA): isolated nasal bridge involvement. Cureus.

[4] Jennette J.C., Falk R.J., Bacon P.A., Basu N., Cid M.C., Ferrario F. (2013). 2012 revised International Chapel Hill Consensus Conference Nomenclature of Vasculitides. Arthritis Rheum.

[5] Cotch M.F., Hoffman G.S., Yerg D.E., Kaufman G.I., Targonski P., Kaslow R.A. (1996). The epidemiology of Wegener's granulomatosis. Estimates of the five-year period prevalence, annual mortality, and geographic disease distribution from population-based data sources. Arthritis Rheum: Off J Am Coll Rheumatol.

[6] Hewins P., Tervaert J.W.C., Savage C.O., Kallenberg C.G. (2000). Is Wegener’s granulomatosis an autoimmune disease?. Curr Opin Rheumatol.

[7] Comarmond C., Cacoub P. (2014). Granulomatosis with polyangiitis (Wegener): clinical aspects and treatment. Autoimmun Rev.

[8] Ibrahim Sumaily I.S., Hussein Etwidy H.E., Daefullah Al-Amry D.A.A. (2018). Granulomatosis with polyangiitis presented as isolated destructive nasal mass-case report. Case Repo. in Otolaryngol..

[9] Ralli M., D’Aguanno V., Falasca V., Turchetta R., Greco A., de Vincentiis M. (2018). Nasal manifestations in granulomatosis with polyangiitis: a case report and review of the literature. Otolaryngol–Open J.

[10] Sasawaki M., Omura K., Ebihara T., Otori N. (2022). A case of granulomatosis with polyangiitis (GPA) where a multicystic nasal septal abscess aided in the diagnosis. Case Rep Otolaryngol.

[11] Maqbool U., Maqbool A., Maqbool A., Qadeer A., Mehmood M.F., Loon M. (2023). An atypical presentation of granulomatosis with polyangiitis: a case report. Radiol Case Rep.

[12] Mehtari NSCS (2024). ENT manifestations revealing Wegener's granulomatosis: case report. J Curr Med Res Opin.

[13] Rusu B., Musat G., Sarafoleanu C. (2025). Rhinosinusal involvement in granulomatosis with polyangiitis–clinical insights. Rom J Rhinol.

[14] Spanuchart I., Zungsontiporn N., Pichaya O., Changcharoen B., Bolger D.T. (2015). Granulomatosis with polyangiitis: a case of nasal mass, necrotic lung, and normal kidneys. Hawai'i J Med Public Health.

[15] Mukhtyar C., Guillevin L., Cid M.C., Dasgupta B., de Groot K., Gross W. (2009). EULAR recommendations for the management of primary small and medium vessel vasculitis. Ann Rheum Dis.

[16] D'Anza B., Langford C.A., Sindwani R. (2017). Sinonasal imaging findings in granulomatosis with polyangiitis (Wegener granulomatosis): a systematic review. Am J Rhinol Allergy.

[17] Tateyama K., Umemoto S., Iwano S., Hirano T., Suzuki M. (2024). Sinonasal manifestations of granulomatosis with polyangiitis: a retrospective analysis. Auris Nasus Larynx.

[18] Wojciechowska J., KręCicki T. (2018). Clinical characteristics of patients with granulomatosis with polyangiitis and microscopic polyangiitis in ENT practice: a comparative analysis. Acta Otorhinolaryngol Ital.

[19] Miloslavsky E.M., Specks U., Merkel P.A., Seo P., Spiera R., Langford C.A. (2015). Outcomes of nonsevere relapses in antineutrophil cytoplasmic antibody-associated vasculitis treated with glucocorticoids. Arthritis Rheumatol (Hob NJ).

[20] Gopaluni S., Jayne D. (2016). Clinical trials in vasculitis. Curr Treat Options Rheumatol.

[21] Stone J.H., Merkel P.A., Spiera R., Seo P., Langford C.A., Hoffman G.S. (2010). Rituximab versus cyclophosphamide for ANCA-associated vasculitis. N Engl J Med.

